# *Notes from the Field:* Asthma-Associated Emergency Department Visits During a Wildfire Smoke Event — New York, June 2023

**DOI:** 10.15585/mmwr.mm7234a6

**Published:** 2023-08-25

**Authors:** Haillie C. Meek, Heather Aydin-Ghormoz, Kathleen Bush, Neil Muscatiello, Cristin E. McArdle, Charlene X. Weng, Dina Hoefer, Wan-Hsiang Hsu, Eli S. Rosenberg

**Affiliations:** ^1^New York State Department of Health; ^2^Epidemic Intelligence Service, CDC; ^3^Division of Environmental Health Science and Practice, National Center for Environmental Health, CDC; ^4^Department of Epidemiology and Biostatistics, University at Albany School of Public Health, State University of New York, Rensselaer, New York.

During June 6–8, 2023, smoke from Eastern Canadian wildfires caused poor air quality across New York, driven by concentrations of particulate matter with aerodynamic diameter ≤2.5 *µ*m (PM_2.5_)*; air quality index reached “unhealthy” or “very unhealthy” levels across the state.^†^ PM_2.5_ from wildfire smoke is associated with an increased risk for medical emergencies, including asthma exacerbations (*1*). Characterizing such health outcomes during this wildfire smoke event can guide current and future response efforts.

## Investigations and Outcomes

Daily mean PM_2.5_ values were calculated using hourly measured concentrations (in *µ*g/m^3^) from one New York State Department of Environmental Conservation^§^ air monitor in each of eight regions^¶^ during June 1–14.** Asthma-associated emergency department (ED) visits were identified from chief complaints in the New York State Department of Health’s Electronic Syndromic Surveillance System (ESSS), capturing all 134 EDs in New York, excluding New York City (NYC).^††^ Daily mean PM_2.5_ concentration was compared with a PM_2.5_ 10-year baseline (2013–2022) for June.^§§^ Daily asthma-associated ED visits were compared between the mean of June 1–5 and June 7, 2023, stratified by region and age group.^¶¶^ June Pearson’s 1–14 correlation coefficients (rho[ρ]) between paired daily mean PM_2.5_ and daily asthma-associated ED visits for each region were estimated. This activity was reviewed by CDC and was conducted consistent with applicable federal law and CDC policy.***

During June 1–14, daily mean PM_2.5_ was highest on June 7 for all regions, except the Adirondacks,^†††^ ranging from 55.2 *µ*g/m^3^ (Western) to 122.3 *µ*g/m^3^ (NYC metro), representing 590% and 1,229% increases, respectively, above 10-year baseline concentrations (8.0 *µ*g/m^3^ in Western and 9.2 *µ*g/m^3^ in NYC metro). During June 1–14, a total of 1,310 asthma-associated ED visits were identified through ESSS (Figure). Compared with the mean number of ED visits during June 1–5, asthma-associated ED visits on June 7 increased 81.9% (from 80.8 to 147 visits) statewide and at least 35.4% for all regions except the Adirondacks.^§§§^ In those regions, the June 1–14 PM_2.5_ and asthma-associated ED visit ρ ranged from 0.31 (Western) to 0.80 (Central). The largest region-specific increases in asthma-associated ED visits and highest ρ estimates were in the Eastern Lake Ontario (179.1% [from 8.6 to 24.0 visits], ρ = 0.70), Central (132.8% [from 11.6 to 27.0 visits], ρ = 0.80), and Upper Hudson Valley (86.4% [from 11.8 to 22.0 visits], ρ = 0.68) regions (Figure). Among persons aged 10–29, 30–49, 50–69, and ≥70 years, statewide asthma-associated ED visits increased 197.6% (from 16.8 to 50.0 visits), 77.1% (from 19.2 to 34.0 visits), 89.0% (from 16.4 to 31.0 visits), and 76.5% (from 6.8 to 12.0 visits), respectively, and decreased 7.4% (from 21.6 to 20.0 visits) for persons aged 0–9 years, from the June 1–5 mean to June 7.^¶¶¶^

**FIGURE:**
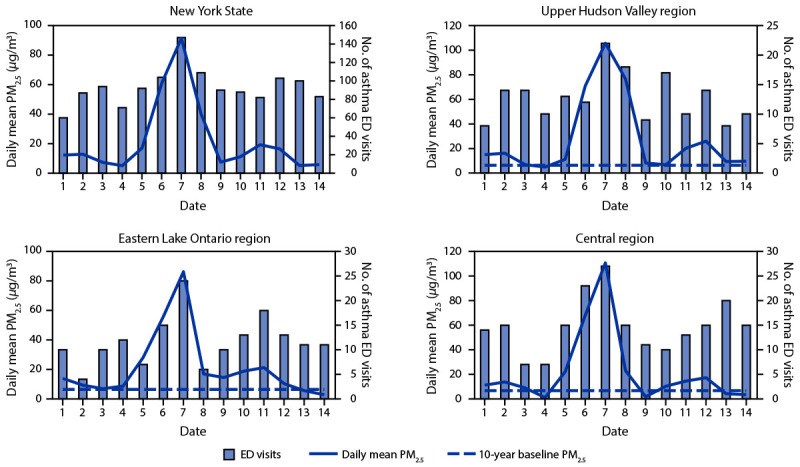
Daily mean particulate matter with aerodynamic diameter ≤2.5 *μ*m and number of asthma-associated emergency department visits statewide* and selected regions^† ^— New York excluding New York City, June 1–14, 2023 **Abbreviations:** ED = emergency department; PM_2.5_ = particulate matter with aerodynamic diameter ≤2.5 *μ*m. * Statewide mean PM_2.5_ based on the region-specific daily mean from each of the eight air quality regions. ^†^ Selected regions had the largest increases in June 7 asthma-associated ED visits compared with the mean during June 1–5.

## Preliminary Conclusions and Actions

During this wildfire smoke event, increased concentration of PM_2.5_ was linked to increased asthma-associated ED visits across New York, with twofold increases in the Eastern Lake Ontario and Central regions and a nearly threefold increase among older children and young adults. Limitations included the attribution of one air quality monitor to an entire region, potential underreporting of asthma exacerbations, and limited covariate data; however, these metrics represent excellent regional, near real-time data, which supported response efforts including recommendations to limit outdoor activities (*2*).

As wildfire smoke events become more frequent and widespread, the findings from this analysis can enhance risk communication and better focus response efforts toward persons at increased risk for asthma exacerbations (*2*,*3*). Children and non-Hispanic Black or African American persons disproportionately experience asthma exacerbations necessitating emergency care****; extreme weather events might worsen these health inequities (*4*). It is essential that public health responses prioritize strategies that reach these populations and promote health equity (*5**)*. These strategies include collaboration with physicians to ensure proactive communication about the risks of wildfire smoke to their patients with asthma and with schools to ensure effective wildfire smoke response plans.

## Footnotes

*
https://www.epa.gov/newsreleases/epa-statement-wildfire-smoke

† Air quality index is a measure that reflects the concentration of five major air pollutants regulated by the Clean Air Act and is based on the health-based national ambient air quality standard for each pollutant. During June 6–7, 2023, PM_2.5_ was the pollutant of concern for all regions. https://www.airnow.gov/state/?name=new-york

§ Data from one monitor per region were used. Nonoptical monitors were preferred; however, in two regions only optical monitors were available. For the New York City (NYC) metro region, a non-NYC monitor was chosen because ED visit data were only for non-NYC counties. Where more than one monitor was eligible, selection was based on data completeness, mean and maximum daily PM_2.5_ concentration, and centrality of location. A monitor in Westchester County was selected for NYC metro region because NYC was not included in this analysis.

¶ New York State Department of Environmental Conservation Air Quality Regions: *Long Island:* Nassau and Suffolk counties; *NYC metro:* Rockland and Westchester counties; *Lower Hudson:* Dutchess, Orange, Putnam, Sullivan, and Ulster counties; *Upper Hudson:* Albany, Columbia, Fulton, Greene, Montgomery, Rensselaer, Saratoga, Schenectady, Schoharie, and Washington counties; *Adirondacks:* Clinton, Essex, Franklin, Hamilton, Lewis, St. Lawrence, and Warren counties; *Eastern Lake Ontario:* Jefferson, Monroe, Oswego, and Wayne counties; *Central:* Allegany, Broome, Cayuga, Chemung, Chenango, Cortland, Delaware, Herkimer, Livingston, Madison, Oneida, Onondaga, Ontario, Otsego, Schuyler, Seneca, Steuben, Tioga, Tompkins, and Yates counties; *Western:* Cattaraugus, Chautauqua, Erie, Genesee, Niagara, Orleans, and Wyoming counties.

** NYC was not included in this analysis. Data collected during June 1–14 were analyzed to characterize 2 weeks of data with ≥5 days of air quality index <150 in all regions before and after the smoke event (June 6–8).

†† Chief complaint search terms were “asthma” and “airway,” excluding “foreign” and “wheeze.”

§§ Ten-year climatological PM_2.5_ baseline levels were tabulated for each region on a monthly basis by New York State Department of Environmental Conservation.

¶¶ Comparisons were made with June 1–5, the period before the wildfire smoke event began and air quality reached unhealthy levels. Patients were assigned to region based on hospital location. Analyses excluded 31 asthma-associated ED visits that were missing hospital data.

*** 45 C.F.R. part 46, 21 C.F.R. part 56; 42 U.S.C. Sect. 241(d); 5 U.S.C. Sect. 552a; 44 U.S.C. Sect. 3501 et seq.

††† In the Adirondacks region, the highest daily mean PM_2.5_ concentration occurred on June 6.

§§§ In the Adirondacks region, compared to the June 1–5 daily mean, June 7 asthma-associated ED visits decreased by 16.7% (2.4 to 2 visits).

¶¶¶ In the Adirondacks region, ρ for daily mean PM_2.5_ and asthma-associated ED visits on June 7 was −0.42.

**** https://www.cdc.gov/asthma/data-visualizations/default.htm (Accessed August 9, 2023).
